# Unfiltered Access, Unseen Harms: A Developmental and Public Health Critique of Digital Rights Discourse

**DOI:** 10.3390/ijerph23030364

**Published:** 2026-03-12

**Authors:** Danielle A. Einstein, Samantha Marsh, Michoel L. Moshel, Talia Sinani, Tracy Burrell

**Affiliations:** 1 School of Psychological Sciences, Faculty of Medicine, Health and Human Sciences, Macquarie University, Sydney, NSW 2109, Australia; 2 Department of General Practice and Primary Health Care, School of Population Health, The University of Auckland, Auckland 1023, New Zealand; sam.marsh@auckland.ac.nz; 3 School of Psychology, Faculty of Science, University of New South Wales, Sydney, NSW 2052, Australia; 4 Clinical Psychology Private Practice, Sydney, NSW 2000, Australia

**Keywords:** child development, adolescents, digital media, social media, public health, digital rights, developmental psychology, screen use, tolerance of uncertainty

## Abstract

**Highlights:**

**Public health relevance—How does this work relate to a public health issue?**

- Around the world, experts are grappling with how to provide guidance for the use of technology for recreational and educational pursuits.

**Public health significance—Why is this work of significance to public health?**

- Rights-based framings of digital access often under-prioritise developmental vulnerability, and fail to recognise the potential for long-term impact on youth.

**Public health implications—What are the key implications or messages for practitioners, policy makers and/or researchers in public health?**

- Children’s digital participation should be nested within a multi-layered public-health response.

**Abstract:**

This article maps and prioritises the foundational developmental needs of children and adolescents: social development, cognitive growth, emotional regulation, identity formation, and moral reasoning. Early and excessive digital engagement is then examined for its potential to impact these milestones, with consequences that reverberate through wellbeing, relationships, and lifelong resilience. Arguments which frame digital engagement as an individual right with potential benefits, downplay developmental risks. Drawing on developmental rights and agency frameworks, the current review disputes the prevailing assumption that digital participation should take precedence over healthy developmental trajectories. Instead, the debate is reframed around children’s evolving capacities. It is proposed that digital entitlements are nested within age-appropriate limits and supports. Protecting the best interests of the child requires recognising the risk of addictive technology use. The rights of the child must also ensure cultivation of emotional competence and self-reliance. Overemphasis on digital expression risks elevating performative self-presentation before moral reasoning, critical thinking, and offline social skills have matured, particularly within environments shaped by algorithmic amplification, transient relationships, peer harassment, and the desire for validation. To address these risks, we advocate for a multi-layered public health response: consistent, developmentally attuned messaging; empowered parents and educators; whole-school strategies; and policy reforms that prioritise safety, accountability, and developmental alignment. By situating digital engagement within a developmental framework, this article proposes key principles on which to base the discussion of safeguarding youth wellbeing in the digital era.

## 1. Introduction

Digital technologies are deeply embedded in the daily lives of most children and adolescents. While the advantages of digital technologies for communication and convenience are frequently emphasised, they are often prioritised over developmental consequences. The ways in which early and sustained digital engagement interacts with core developmental processes (e.g., social, neural, and cognitive) are often underexplored. A growing body of evidence points to concerns across multiple domains, including toddler language development [1], neural maturation, brain organisation [2,3], cognitive functioning [4,5,6], emotion regulation capacities [7,8], and social development, where reduced in-person interaction and heightened online comparison appear especially influential [9,10]. Research also indicates that increased reliance on digital platforms for belonging may contribute to compulsive or addictive patterns of internet and social media use [11,12].

In 2025, two influential articles acknowledged many of these harms while proposing that social media may also deliver benefits for youth and adolescents [13,14]. In this article, we argue that identified benefits can be facilitated within moderated interest groups, through access to the internet, and through 1:1 or 1-to-small-group communication with identified others.

We start this review by referring briefly to the topics on which there is agreement. The risks for mental health are:

- Exposure to cyberbullying and unwanted online sexual solicitation (both of which are consistently linked to higher rates of depression, self-harm, and suicidal behaviour in adolescents [15,16,17].
- Exposure to harmful content and communities, including pro-eating disorder material and appearance-focused ideals that negatively impact body image, particularly among girls [13,18].
- Time spent on smartphones and social media is observed to be associated with poor mental health, reduced physical activity, reduced cognitive ability, and sleep disruption [14,19,20].
  We add:
- Radicalisation, aggression, rising loneliness, lowered self-esteem, and increased emotional fragility in late adolescence [21,22,23,24].

In Australia, rates of adolescent depression have risen sharply (from approximately one in five young people meeting criteria for depression by age 18 to nearly two in three in recent cohorts), mirroring international trends of worsening youth mental health [22,25,26,27]. Moreover, the chronic nature of these difficulties is especially pronounced among girls, roughly half of whom follow persistent depressive courses during adolescence [28], making this developmental period increasingly challenging. We argue that the scale and urgency of mental health problems are, in part, caused by the failure of industry, researchers, and policy advisers to consider how development is interfered with by current digital offerings.

The broader discourse surrounding youth digital use and mental health has become highly contested. Rights-based framings, such as those advanced in the BMJ and earlier commentaries, sometimes underplay the developmental vulnerabilities that shape how young people engage with digital environments [29,30]. Simultaneously, an expanding industry has emerged to address the mental-health problems that appear linked, in part, to intensive or developmentally misaligned technology use. Evidence consistently shows that high levels of smartphone and social-media engagement are associated with depressive symptoms, anxiety, and sleep disturbance [31,32], indicating that downstream interventions are unlikely to be sufficient on their own.

This review seeks to break down the key arguments that have been put forward, which may stifle a multi-layered approach to public health [33]. From a public-health perspective, consistent, developmentally informed messaging is essential to guide community attitudes and support parents in delaying, reducing, or reshaping digital media consumption. Parenting practices (e.g., active mediation, limit-setting, and supportive co-use) may reduce problematic digital engagement [34,35,36,37]. Active mediation (the parent discussing media-related concerns without criticism) and consistent limit-setting appear most effective in childhood and early adolescence, whereas for older adolescents, autonomy-supportive practices and parent–child relationship quality are more strongly associated with healthier digital engagement than rule-based restriction alone [34,35]. However, these practices cannot reasonably counteract environments engineered to leverage reward sensitivity and attentional capture, underscoring the need for structural, regulatory, and safety-by-design approaches.

### 1.1. The Primacy of a Child’s Right to Development

This review is grounded in the principle that a child’s right to healthy social, neural, and cognitive development must take precedence over other competing rights. These developmental capacities form the foundation for the later exercise of autonomy, expression, participation, and informed decision-making. When digital engagement occurs early, frequently, or without developmentally appropriate support, it has the potential to interfere with the acquisition of core regulatory, social, and cognitive competencies. The following Table 1 outlines key developmental domains and summarises the mechanisms through which digital environments may disrupt, displace, or distort typical developmental trajectories.

### 1.2. Rethinking the Language of Rights

Children’s rights in digital spaces are typically interpreted through the UN Convention on the Rights of the Child (UNCRC). Yet these frameworks were drafted for a generic rights-bearing subject and often assume a level of maturity, stability, and agency that may not reflect children’s evolving capacities, especially in highly engineered, commercialised online environments. Table 2 reframes key UNCRC articles through a developmental lens, highlighting where standard digital-rights framings overlooked age-related needs and vulnerabilities.

While Table 2 reframes selected UNCRC articles through a developmental lens, the underlying right to development itself remains comparatively under-specified in digital-rights debates. Table 3 contrasts a rights-based framing of children’s digital engagement with a developmental framework, highlighting how the right to development is often under-prioritised or only superficially addressed.

### 1.3. Children’s Agency and Access to Accurate Information

Children are agents in their own digital lives, but meaningful agency depends on access to accurate, evidence-based information. In current debates, the language of digital “benefits” often overstates advantages that are tangential to mental health and can become detrimental when over-relied upon. At the same time, this framing can misconstrue or downplay documented harms. Table 4 illustrates how commonly cited digital “benefits” may be partial, misleading, or contingent on important caveats.

### 1.4. The Role and Limitations of Digital Literacy Programmes

Digital literacy education is now a central plank of school-based responses to online risk. These programmes aim to equip young people with skills to evaluate digital content, recognise cyberbullying and manipulative design, and make more informed choices about their online behaviour. However, evaluations of widely implemented curricula (e.g., cyberbullying and social media literacy programmes) suggest that gains are often modest, uneven across subgroups, and short-lived, with limited evidence for sustained improvements in behaviour or wellbeing. Evaluations are often based on measures of knowledge from the training. Short program duration, reliance on self-report, insufficient integration into whole-school practice, and minimal involvement of parents or wider community systems all constrain their impact.

While digital literacy must remain part of a multi-layered public health response, it cannot, on its own, safeguard children’s development in commercialised, attention-driven digital environments. Over-reliance on these programmes risks creating a false sense of assurance and shifting responsibility onto children to navigate systems that remain fundamentally misaligned with their developmental needs. Table 5 summarises the intended role of digital literacy initiatives, the key limitations that emerge from current evidence, and the implications for policy and practice.

## 2. Discussion

Having reviewed the limitations in a “rights” based approach (Table 3) via the vehicles of development (Table 1), agency and digital literacy education (Table 2, Table 3, Table 4 and Table 5), the discussion turns to a theoretical exploration of essential psychological concepts that should underpin a public health approach. This discussion explores the cascading effect of addictive design on self-regulation, modelling, and mental health.

### Recognising the Addictive Pull and How It Expands to the Environment

The device functions as a conditioned stimulus: its sensory cues (e.g., vibration, screen illumination) are repeatedly paired with social feedback delivered on variable schedules, strengthening cue-reward associations (i.e., via Pavlovian and operant mechanisms) [144,145]. Over time, the cue alone can elicit physiological arousal, attentional capture, and approach tendencies, much as notification studies show an increase in inattention and urge to check when phones signal or are merely present [146,147]. Additionally, contextual features (e.g., bedrooms, desks, evening hours) acquire associative value that further facilitates engagement [148]. Based on this, the pull to re-engage with devices reflects predictable conditioning and reinforcement processes described in contemporary models of problematic smartphone use, rather than a simple moral failing or lack of willpower. The ‘pull’ expands to bedrooms, homes, and classrooms.

We propose that modern digital environments interact with adolescents’ heightened sensitivity to rewards and social evaluation, reweighting how goals, motivation, and uncertainty are managed [74,76,149]. Social media platforms leverage learned anticipation and peer feedback, encouraging frequent and constant engagement [150,151,152,153].

Contemporary revisions challenge Maslow’s original sequencing of needs, treating belonging as a fundamental, evolutionarily grounded motive rather than a “higher-order” luxury. Baumeister and Leary’s classic need-to-belong account argues that humans strive to form and maintain close bonds with an urgency comparable to basic drives [154], a view echoed in more recent syntheses of belonging and wellbeing [155]. Kenrick et al. likewise restructure Maslow’s pyramid, proposing a dynamic hierarchy in which motives for affiliation and status can be prioritised alongside survival concerns [156].

In device-saturated contexts, these social needs are not only basic but continuously cued. Social platforms repeatedly signal potential gains and losses in inclusion, status, and connection; the need to belong and fear of missing out reliably predict problematic smartphone use, especially among youth high in intolerance of uncertainty [12]. Technology-based social comparison and feedback-seeking prospectively forecast increases in depressive symptoms [9], while young people frequently report using digital media to distract from distress, to seek reassurance, and to meet perceived external standards of perfection [157,158]. Together, these behaviours mark a broader psychological shift toward restlessness, urgency, and diminished tolerance for uncertainty. Tolerance of uncertainty has been shown to protect against the development of social anxiety, panic disorder, and generalised anxiety disorder in adolescence [159]. Avoiding uncertainty drives maladaptive emotion regulation mechanisms. It must be prioritised in prevention efforts that target digital media use. Altering this factor has been shown to have a limited impact on adolescent social media use [92,160,161].

Importantly, the “addictive” pull should be distinguished from clinical addiction, with most cases better framed as conditioned responding and uncertainty-driven checking, even though a non-trivial subset may progress toward genuinely addictive patterns with prevalence varying between 18.5% and 23.3% in reports globally [11,162,163,164]. Acknowledging the ‘addictive pull’ may prompt self-regulation by adults in the home environment. This, in turn, will assist parents in modelling healthy boundaries around technology use. We argue that this information should be provided within public health education. A broad understanding of these concepts will assist leaders (be they educators, librarians, parents, or other decision makers) to design helpful practices and changes to the environments that they oversee. We have already seen initiatives commence within phone-free schools, phone-free restaurants, and phone-free camps. Similarly, tech entrepreneurs are reported to deliberately delay the provision of devices for children. In a world where technology is introduced to children by both schools and parents, it is essential to recognise the harms brought about by the addictive use of technology.

Emerging reviews and policy reports argue that bolstering self-regulation under such continuous stimulation will require coordinated policy, digital-literacy education, and family and school-level practices [45,165,166,167,168].

## 3. Features Within a Multi-Layered Approach

Recent technological innovations have flourished in a world with a lack of recognition of the adverse developmental impact of their offerings. In this environment, no single intervention or stakeholder acting alone can protect youth mental health. Because digital environments now function as a core social determinant of health with commercial interests driving use, a coordinated, multi-layered response is required [127,169]. The denial of harms to child development, emotion regulation, and community cohesion has prevented the systems around children from leading with thoughtful guidelines. Elements of a multilayered approach which could be considered and trialled are set out below.

(1) Regulation: Developmentally informed regulation might include the following:
  (a) Minimum-age provisions which place the onus on platforms to age-gate entry of users effectively;
  (b) Duty-of-care laws which require platforms to take care to prevent harms experienced from engagement via their platform;
  (c) Safety by design principles which remove the inclusion of likes, comments, streaks, autoplay, pull-to-refresh, recommendation systems, companion chatbots, randomised gaming features (e.g., in-app currencies, reward loops, fortune wheels, pay-to-progress) which aim to engage the user for longer time periods and play on the self-worth of the user; ban advertising for minors;
  (d) Privacy regulation to ensure that data from underage users is not collected;
  (e) Removal of anonymous posting to increase the sense of responsibility towards amplifying emotive content and/or misinformation.
  The EU Members of Parliament recommended that senior managers within Technology companies be made personally liable in cases of serious and persistent non-compliance with regulation [170].
(2) Adult Education:
  (a) Public health education should recognise the ‘addictive pull’ of portable devices. This will normalise new practices recommended in recommendations 3 and 4.
  (b) Education for prenatal parents to understand the impact of parental and infant technology use in the early years. Parenting education that supports parents with alternative means of managing challenging behaviours in the early years.
(3) Environmental Changes (through either regulation or policy guidelines led by overseeing systems):
  (a) Within Schools: Initiatives spurred on by Australia’s regulation—school guidelines requiring students not travel to or from school with smartphones, boarding school access to phones limited to 30 min per day, removal of QR codes from signs around schools. In the United Kingdom, the Department of Education has released a new phone policy with detailed guidelines, including suggestions for navigating concerns for students with special needs. Notably, computers provide similar communication features, and consideration of initiatives may need to expand to school-enabled devices.
  (b) Within Public Places: device-free playgrounds, libraries, and restaurants. These occur due to the foresight of a council, owner, or board that wishes to support such practices.
  (c) Technology programmes used within school systems should meet a minimum threshold by obtaining an independent agency rating that confirms the absence of gamification elements. Metrics of engagement must be distinguished from metrics that demonstrate genuine learning outcomes.
(4) Secondary Prevention: Professional development for medical practitioners, clinicians, and psychologists to assist them in assessing when device use maintains psychological symptoms. Clinical guidelines for these professionals.
(5) Educational initiatives for youth need to address two critical areas.
  (a) Foster digital literacy skills through developmentally attuned messaging to protect against the harms young people may encounter when using the internet. These must be subject to independent evaluation to justify investment of time and resources. Although digital literacy is frequently promoted as a “primary prevention strategy”, without sustained reinforcement, active parental involvement, and broader community engagement, programmes have the potential to be counterproductive if they act as symbolic gestures and do not lead to actual behaviour change. To date, the empirical evidence base shows mixed outcomes. The Safe-Surfing program (covered harassment, passwords, malware, social media) and found only small, short-term, and non-universal improvements [138,139,140]. SoMe (four-session social media literacy program) showed no meaningful behaviour change [143]. Meta-analyses of whole school bullying programmes show approximately a 15% reduction in bullying across schools, leaving many students still targeted after interventions [171].
  (b) Co-design of initiatives that support adolescents to reduce addictive patterns of social media and technology use. With regard to opinions on social media use, in New Zealand, at the end of 2025, 540 youth were surveyed (aged 13 to 17; 61% male). In this survey, 39% of the adolescents stated that they “wish social media had never been invented”. In this group, 47% supported the age restriction of 16 years, 25% opposed an age restriction, while the remainder stated that they did not care. Finally, 32% of teens reported it would be easy to give up social media, but this proportion almost doubled (61%) when teens felt that their friends would give it up as well. These findings highlight the impact of social pressure on the decisions that are being faced [163]. It may be that, as vulnerable groups work through challenges, they explore the benefits of one-to-one or small-group communication with identifiable others rather than relying on large public platforms. Such one-to-one communication may reduce social signalling demands and performative pressures within discussion.
  (c) In changing norms, it may also be helpful to seek leaders in popular culture who endorse the changes and who call out the problems that are seen in youth vulnerabilities being exploited.

### Future Directions

Future research should establish a clear framework that first distinguishes meaningful learning and personal productivity from product engagement. Such a framework should weigh these distinctions against the practical benefits of digital use for individuals and peer groups at different developmental stages. Future work should also further explore gender differences pertaining to each developmental area and the harms that children and young adolescents are exposed to via social media. It is timely to frame children’s digital participation within a multi-layered public health approach. This means that every part of the community will work together to accurately recognise harms and put small initiatives in place. The rights-based perspective must recognise key developmental principles set out here. This will ensure that every child’s right to healthy development is protected from addictive social forces that impair emotional, social, and cognitive functioning.

## Figures and Tables

**Table 1:** Mechanisms Through Which Digital Environments Interact with and Disrupt Key Developmental Domains.

Developmental Domain and Priorities of Relevance	Proposed Mechanisms	Outcomes
Social Development Across childhood and adolescence, social development involves acquiring foundational interpersonal skills (e.g., eye contact, turn-taking, conversational reciprocity), progressing to group navigation, perspective-taking, and stable peer relationships [38,39]. These competencies require repeated offline practice, social feedback, and real-time conflict resolution [40,41,42]. *Age of relevance:* Children and Adolescents	**Cyberbullying and Social-Evaluative Threat** Online peer spaces can expose children and adolescents to persistent (often anonymous) harassment, public ridicule, and cyberbullying at large scales [43]. In Australian data, a small sample of trans and gender-diverse youth were identified, 69% had experienced cyberbullying in the previous 12 months [44].	Cyberbullying is more common amongst teens than younger children [45]. Cyberbullying victimisation is consistently associated with poorer mental health, lower perceived safety, greater unhappiness and, in more severe cases, self-harm and suicidal ideation [45,46,47]. Girls are more likely to be cyberbullied than boys.
	**Amplified Social Comparison and Validation-Seeking** Social media design affords continuous exposure to idealised peer content (appearance, popularity, lifestyle) alongside quantifiable feedback (likes, comments, followers), reinforcing upward comparison and validation-seeking [9].	Meta-analytic and experience-sampling evidence links exposure to idealised content and feedback-contingent engagement to negative self-evaluations, fluctuating self-esteem, and increased risk for depressive and anxiety symptoms, particularly on high-use days [48,49,50].
	**Displacement and Restructuring of Social Time** High daily screen and social media use displaces face-to-face interaction and practice of social problem-solving, negotiation, and emotional attunement [51].	Heavy personal screen time (e.g., ≥7 h/day) and passive scrolling beyond ~2 h/weekday are associated with reduced offline socialising and elevated odds of clinical anxiety, depression, psychological distress, and suicidal ideation [51,52].
	**Influencer and Parasocial Impacts on Norms and Identity** Influencers act as models for adolescents [53,54]	Influencer exposure shapes perceived norms, risk behaviours, and identity aspirations, with emerging evidence that it can both reinforce harmful ideals (e.g., thinness, substance use) and alter health-related decision-making [53,54].
Cognitive Development Cognitive development is driven by the maturation of several interrelated systems: sustained and selective attention, executive functions (e.g., inhibitory control, cognitive flexibility, working memory, etc.), decision-making, language and literacy acquisition, and mentalising or theory-of-mind capacities [4,55,56]. *Age of relevance:* These systems undergo rapid change during middle childhood and early adolescence.	**Stimuli and Attentional Dysregulation** Fast-paced, high-intensity, and reward-rich digital content engages bottom-up attention and encourages frequent task switching [4,57,58].	Such exposure is linked to distractibility, poorer sustained attention, and academic difficulties [4,6].
	**Reduced Language Input and Literacy Practice** Visually dominant or fast-paced media often replace reciprocal conversation, shared reading, and structured linguistic input essential for language and literacy development [59].	Early screen exposure is associated with poorer longitudinal development of executive function in toddlerhood [55]. Because executive functions support sustained attention and self-regulation during parent–child interaction and shared reading, early reductions in these capacities may indirectly limit children’s engagement in language-rich and literacy-building activities [60].
	**Memory and Decision-Making Declines** High exposure to fast-paced and cognitively fragmented digital media may interfere with the maturation of frontoparietal networks that support working memory, inhibitory control, and goal-directed decision making [61,62].	Neuroimaging studies report reduced grey-matter volume and functional differences in executive regions among heavy users [2,6], with associated deficits in memory, decision-making, and problem solving that correlate with higher depressive symptoms and academic impairment [2,63].
	**Impaired Self-Regulation and Reward Sensitivity** Variable-reward notifications, infinite scroll, and rapid engagement cues strengthen habitual checking and undermine opportunities to practise delaying gratification [64,65,66].	These patterns predict higher impulsivity, weaker self-regulatory control, and greater risk of problematic digital-use behaviours, which are associated with elevated stress and internalising symptoms [64,65].
	**Sleep Disruption from Night-Time Device Use** Evening use of devices exposes children and adolescents to blue light, social notifications, and emotionally arousing content, delaying circadian rhythms and reducing sleep duration and quality [67,68,69].	Sleep disruption predicts poorer executive function, emotional reactivity, irritability, and increased depressive symptoms [68].
	**Cognitive Overload and Multi-Tasking Demands** Constant task switching, parallel app use, and continuous notification streams impose chronic cognitive overload and exceed working-memory capacity [70,71].	Cognitive overload is associated with fatigue, stress, and reduced performance on tasks requiring sustained attention or planning [71]. Longitudinal data show that even low levels of regular social media use predict measurable declines in cognitive performance over two years, with heavier use linked to larger impairments [5].
Neural Development Adolescent brain development is marked by heightened sensitivity of reward and socioemotional circuits (e.g., ventral striatum, amygdala, insula) and more gradual maturation of prefrontal control networks. This imbalance leaves early adolescents particularly responsive to peer and reward cues while self-regulatory systems are still consolidating [72,73,74,75]. *Age of vulnerability:* Early adolescence	**Heightened Neural Sensitivity to Social Feedback and Rewards** Social media exposes adolescents to dense streams of likes, comments, and status cues precisely when neural circuits for social reward and evaluation are highly plastic [76]. Complementary longitudinal MRI work links habitual checking with increasing activation in reward and social-evaluation regions during anticipation of feedback [50].	This heightened sensitivity is associated with stronger peer influence on mood and risk-taking and greater emotional impact of online exclusion or status loss. This increases vulnerability to anxiety, depressive symptoms, and reputation-driven risk behaviours [72,76,77].
	**Dopamine and Habit Formation** Variable, intermittent rewards (e.g., unpredictable notifications, intermittent social validation) recruit mesolimbic dopamine circuits. Repeated activation induces molecular plasticity, including ΔFosB accumulation and altered spine density and glutamatergic signalling in nucleus accumbens neurons. These adaptations bias behaviour toward cue–response habits and more compulsive patterns of digital checking [78,79].	Behaviourally, this manifests as problematic social media use (preoccupation, failed cut-down attempts, use despite harm), which is consistently linked to higher depression, anxiety, stress, and everyday cognitive failures [63,80,81].
	**Altered Functional Connectivity in Heavy/Problematic Use** Neuroimaging in adolescents with problematic digital use shows altered connectivity across default mode, executive-control, salience, and reward networks, including reduced fronto-striatal connectivity and disruptions in attention and self-referential networks [4,64,82,83,84].	These connectivity and executive-function alterations are linked to higher rates of internalising symptoms, stress, and impaired daily functioning among adolescents with problematic digital use, including more severe anxiety, depressive symptoms, and emotion-regulation difficulties [63,81,85].
Emotional Regulation Learning to soothe frustration, tolerate boredom, and manage uncertainty without constant external input are key facets of emotional regulation. These skills develop through repeated experiences of waiting, wondering, self-soothing, and problem-solving in everyday situations. *Age of vulnerability:* Adolescence	**Digital Emotion Regulation and Calming Meltdowns** Parents increasingly use phones or tablets to stop meltdowns or keep young children calm in challenging settings, displacing opportunities to practise natural emotion-regulation strategies and effortful control [86,87].	Longitudinal studies show that screen-based soothing predicts greater emotional reactivity, poorer executive functioning, and weaker effortful control; in temperamentally angry children, a bidirectional cycle emerges in which dysregulation and tablet use reinforce each other [86,88].
	**Boredom Avoidance and Continuous Stimulation** Both using social media out of boredom and using it for social connection are predictors of Problematic Social Media Use [88]. On-demand, high-intensity digital stimulation at the first sign of boredom reduces unstructured time that would otherwise prompt imaginative play, self-directed activities, or reflection [89,90].	Boredom proneness mediates associations between problematic smartphone use and higher depression and anxiety, with clinical presentations often characterised by low frustration tolerance, chronic restlessness, and worsening mood when devices are removed [89,90]. Satisfaction with schoolwork declines with more social media use in boys and girls at each age (10 to 15) [91].
	**Intolerance of Uncertainty and Online Reassurance-Seeking** Social media offers rapid ways to reduce uncertainty (checking views, replies, or status) while simultaneously generating new ambiguities (e.g., delayed responses, ambiguous posts), encouraging reassurance-seeking loops [89,92].	Affectively charged interactions can easily spiral into mental health symptoms such as anxiety and depression. This is particularly true for young people with histories of early and ongoing developmental adversity (ACEs), who are particularly vulnerable to epistemic mistrust and emotionally charged interactions [93].
	**Emotional Reactivity to Social-emotional Cues is amplified in Digital Environments** The social media culture of broadcasting and commenting adds a layer of complexity for adolescents who are immature in their capacity to cognitively regulate their reactions online. Failures to regulate interpersonal responses on social media are shared, commented on, and recorded within apps, creating a social interactional world where all mistakes are exposed and noticed. Unsolicited social media peer feedback requires additional cognitive effort to integrate this information into the adolescents’ understanding of themself and others. The parallel world of the social media environment adds a second layer of complexity and stress to the developing emotion-regulation system.	Brain changes are associated with increased reactivity to emotional cues in the social environment [94,95].
Identity Formation Identity formation involves building a coherent, relatively stable sense of self grounded in internal values, integrating experiences across contexts, and allowing time for solitude, role exploration, and self-reflection [96,97]. Identity formation includes developing real-life skills grounded in activities and experiences that reinforce self-efficacy. Skills and identity develop slowly through experiences of frustration, failure, and setbacks. Overcoming these in the offline environment builds intrinsic motivation and the ability to delay gratification [98]. *Age of Vulnerability:* Adolescence	**Contingent Self-Esteem and Feedback-Driven Identity** Social media platforms frame identity as continuously rated and ranked through likes, comments, and follower counts, encouraging self-worth to become contingent on online reactions [99,100]. Younger adolescents are more likely to present themselves through their abilities, while older adolescents instead define themselves through their opinions [101]. Gamification within online programmes activates the dopamine system and has the potential to undermine self-efficacy and motivation [102].	Adolescents high in social-media-contingent self-esteem show stronger emotional reactions to online criticism and are more likely to curate identities that maximise approval but heighten sensitivity to disapproval; over time, this is associated with lower self-esteem and greater anxiety and depressive symptoms [99,100].
	**Fragmented, Externally Oriented Self-Concept** Opportunities to curate multiple profiles, experiment with discrepant selves, and maintain large networks of weak ties can crowd out offline solitude and reflection and orient identity toward audience approval [96].	Lower self-concept clarity and discrepant online–offline selves are linked to feelings of inauthenticity, social anxiety, self-consciousness, and strain in offline relationships [96].
	**Self-Focused Disclosure and Mental Health as Identity Content** Platforms make high-frequency, intimate self-disclosure and engagement with mental-health narratives easy [103,104]. Psychological symptoms are romanticised, making distress seem desirable (as it increases engagement). This normalises self-diagnosis [105,106,107].	Excessive, self-focused disclosure and identity fusion with distress (“this is who I am”) are linked to greater rumination, higher depressive and anxiety symptoms, and increased problematic social media use, as distress becomes intertwined with engagement and validation-seeking [108,109].
Moral Reasoning Moral reasoning involves developing reflective judgement, empathy, and the ability to weigh context, intentions, and consequences, moving from simple rule-following to nuanced ethical reflection and responsibility-taking.	**Algorithmic Delegation and Ontological Assimilation** Algorithmic systems pre-select what young people see, who they interact with, and which options are “recommended,” shifting moral decision points upstream and encouraging deference to opaque recommendation systems [110,111,112]	Repeated delegation of choice in morally relevant contexts (e.g., news, political content, who to follow) can erode perceived agency and responsibility, fostering helplessness, cynicism about fairness, and disengagement from civic and ethical discussion [113].
	**Emotionalised, Polarising Feeds and Group Mentality** Engagement-driven recommender systems amplify moral-emotional content (outrage, condemnation), particularly within like-minded groups, increasing perceived conflict and moralisation of issues [110,111,114].	Overexposure to others’ outrage heightens perceived intergroup threat and hostility, reinforcing black-and-white thinking, reducing tolerance for ambiguity, and contributing to anxiety, anger, and interpersonal conflict [114].
	**Non-Moral Algorithms Shaping Moral Cues** Algorithms trained on decontextualised data misread nuance (e.g., irony, power imbalance, local norms) and simulate moral judgement without understanding (“moral zombies”), yet still rank, flag, and frame content, thereby shaping what appears morally salient or acceptable [112,115].	Distorted cues about what is harmful or permissible can confuse young people about norms, foster distrust in institutions (“everything is biased”), and increase vulnerability to radicalised or conspiratorial communities offering simple moral narratives, contributing to anger, distrust, and psychological strain, particularly among those already feeling marginalised [116].

**Table 2:** Limitations of standard rights frameworks in addressing children’s developmental needs.

UNCRC Article and Typical Digital-Rights Interpretation	Overlooked Developmental Need	Why This Matters
**Article 2—Non-discrimination** Promotes equal access to digital devices, platforms, and online spaces for all children, emphasising that no child should be excluded from online opportunities on the basis of background or identity [117] (art. 2.).	Assumes that access is uniformly beneficial, overlooking that children at different ages (e.g., with different temperaments, neurodevelopmental profiles, or vulnerabilities) have very different cognitive, emotional, and social capacities to use digital tools safely and productively [10].	Non-discrimination must go beyond equal access to include equitable, developmentally tailored support. Without accounting for evolving capacities, “equal” access can widen disparities in outcomes: younger or more vulnerable children may be more exposed to harm, less able to self-regulate, and more easily manipulated by persuasive design [10,29].
**Article 12—Right to be heard** Children should participate in decisions about their technology use and in digital governance debates, with their online voices respected in policy and platform design [117] (art. 12).	Often assumes children’s views are fully informed and autonomously formed, underplaying their reliance on adult, expert, and evidence-based guidance. It neglects how misinformation, influencer content, and algorithmically amplified personal stories can shape children’s perspectives, sometimes more strongly than balanced, empirical recommendations [29].	Respecting children’s voices requires ensuring they have access to reliable, age-appropriate information and scaffolding to interpret it. Otherwise, “listening to children” may simply mean endorsing narratives which dominate their feeds, rather than supporting the gradual development of informed, reflective judgement [118]. Children have a right to the internet without monetisation and engagement metrics disrupting their voice (see below).
**Article 13—Freedom of expression** Emphasises children’s right to express themselves, share opinions, and create content through digital media, including social platforms, videos, and user-generated content [117] (art.13).	Expression is treated as an unqualified good, with little attention to how constant broadcasting and audience feedback can distort healthy identity formation and emotional maturity. Platform logics favour speed, emotional intensity, and controversy, encouraging urgent posting, reactive commentary, and attention-seeking self-presentation over slow thinking, private reflection, and exploration away from peer surveillance [10].	Expression should be enabled, not exploited. When persuasive design and recommender systems nudge children toward continuous self-display (including romanticised “mental health” content as identity) self-worth risks being built around visibility rather than values. Tech philosopher James Williams argues that systems designed to manipulate attention and choice undermine meaningful agency: without real freedom of attention and decision, freedom of expression becomes hollow [119].
**Article 15—Freedom of association** Frames social media and online communities as vehicles for connection, participation, and inclusion. This is particularly prevalent for marginalised, isolated, or minority youth [117] (art. 15).	Underplays the quality, stability, and developmental appropriateness of online relationships. Structural risks such as exclusion, harassment, comparison, and social anxiety are often treated as incidental. Many online ties are fleeting, shallow, and easily abandoned; yet they can exert more influence than in-person relationships, especially for socially vulnerable young people [10].	Association is only meaningful when children are developmentally prepared to navigate complex, algorithmically shaped social dynamics. If digital ties routinely displace or weaken offline relationships (rather than complementing them), then the right to associate online may erode the very social foundations (secure attachment, stable peer bonds) that support resilience and mental health [10,118,120,121,122]
**Article 17—Access to information** Stresses children’s right to access diverse media, news, educational resources, and online content, including user-generated material [117] (art. 17).	Pays limited attention to how unfiltered, hyper-personalised, or developmentally misaligned content can overwhelm, frighten, or confuse children. It often assumes that more information is inherently empowering, regardless of its emotional intensity, accuracy, or cognitive complexity [29,123].	For children, access must be age-appropriate, scaffolded, and emotionally safe. Developmentally aligned digital literacy (and supportive adult mediation) helps children distinguish credible from misleading sources, cope with distressing material, and integrate information into a coherent understanding rather than anxiety or confusion. Without this, the right to information can become a right to unbuffered exposure in an environment optimised for engagement, not understanding [124].
**Article 24—Right to health** Highlights the benefits of digital tools for mental and physical health, such as online support communities, telehealth, wellbeing apps, and psychoeducational resources, often arguing that these positives justify broad youth access [117] (art. 17.).	Tech-related harms (e.g., anxiety, disrupted sleep, attention dysregulation) are frequently framed as secondary, individual, or inevitable side effects. There is limited focus on how everyday platform design and attention-economy incentives interact with developmental vulnerabilities to shape long-term health trajectories [10,118].	A developmental lens treats digital environments as part of the social determinants of health: chronic sleep loss, attentional overload, and ongoing social-evaluative threat can lay the groundwork for enduring mental-health difficulties [10]. Protecting the right to health, therefore, requires not only preserving beneficial “doors to digital health” (e.g., reputable helplines, evidence-based apps, moderated peer support), but also intentionally designing and regulating systems so these are easy to find and use, while constraining features and business models that systematically undermine emotional regulation, rest, and recovery [124].

**Table 3:** A rights-based approach without a developmental framework.

Rights Principle	Goodyear et al. (2025) Framing [30]	Developmental Focus Present?	Developmental Limitation
**Non-discrimination**	Ensure equal access to meaningful digital environments; promote tolerance and equality	No	Focuses on access/equality, but not whether environments are developmentally appropriate
**Best interests of the child**	Fulfill rights to access, share information, and be protected from online harms	No	Balances access and safety, but does not consider children’s evolving self-regulation or emotional needs.
**Right to development (Article 6)**	Support children’s growth via digital environments to develop knowledge, skills, talents, and abilities	Partial	Cognitive capacities that are disrupted during childhood or adolescence may produce cascading developmental effects. When impulse control or attention regulation systems are compromised, the assumption of a fully autonomous, free-willed user becomes tenuous.
**Right to be heard (Article 12)**	Promote participation and inclusion in digital contexts; support children to express their views online	No	Encourages expression without addressing the capacity to process feedback or handle digital risks.
**Child participation in shaping tech use**	Include children in identifying problems with social media and digital tech	No	Well-intentioned, but assumes children have the developmental insight to critique complex digital systems.

**Table 4:** The language of digital “benefits” can downplay harms.

Claimed Digital “Benefit”	Critical Analysis/Important Caveats	Supporting Source(s)
Social support and connection for minority groups (e.g., LGBTQIA+, Aboriginal and Torres Strait Islander youth, children with disabilities)	While online spaces can offer validation and community, over-reliance on digital connections can reduce in-person interaction and social skills. This, in turn, amplifies feelings of isolation. It has been observed that online-only interactions do not alter the moment ratings of loneliness in youth [125]. Emotional investment in LGBT social media has been negatively associated with well-being. It has been described as a ‘double-edged sword’ associated with benefits and risks for minority groups [126]. Relationships via social media may be non-reciprocal, unstable, or emotionally unsustaining.	Refs. [13,14] Critical reflection/policy debate framing
Increased access to information and mental health resources	Access alone does not equate to benefit. Exposure to unverified, triggering, or overwhelming information can have the opposite effect, especially without evidence-based digital literacy. Misinformation is prolific [127].	[14]
“Connection” through social media use	Describing social media use as “connection” can obscure rising adolescent loneliness. The digitalisation of social interactions has exacerbated loneliness, as young people often reduce face-to-face interactions [128]. Group connection incentivises subtle aggressive behaviours towards those in the ‘out-group’ [129]. Passive or one-sided engagement (scrolling, watching, observing others interact) is not equivalent to mutual, emotionally supportive relationships, and may leave young people feeling more excluded or inadequate.	[14]
Open platforms for expression and belonging	Platforms that enable self-expression and community also provide channels for cyberbullying, hate speech, sexual solicitation, and harmful communities (e.g., pro-eating-disorder forums, appearance-ideal content). Without strong safeguards, the same affordances that support belonging can simultaneously expose young people to serious psychological risks.	[13]
Time spent online as “normal adolescent behaviour”	Framing high screen use as merely normative can normalise risk and mask harm. Across multiple studies, increased time on smartphones and social media is significantly associated with poorer mental health, reduced physical activity, and disrupted sleep.	[14]
Trigger warnings empower informed choice	In practice, 90% of young users approach content that carries a trigger warning. Experimental work shows that generic warnings can increase state anxiety and curiosity, particularly when wording is vague. More specific warnings combined with friction (e.g., clicks, delays, content blurring) are more likely to reduce viewing than labels alone [130,131,132,133,134,135,136].	

**Table 5:** The role and limitations of digital literacy programmes in supporting healthy development.

Proposed Role of Digital Literacy Programmes	Limitations/Challenges	Implications
**Equip children and young people with skills to critically navigate digital spaces** (e.g., recognising cyberbullying, unsafe content, and online risks).	Outcomes on online-safety knowledge are uneven. For example, the peer-led STAnD programme in Budapest improved cyberbullying and internet-safety knowledge and empathy, particularly among girls, but gains were not uniform across students and tended to fade over time [137]. Systematic reviews report that school-based cyberbullying programmes generally show stronger effects on media skills (e.g., recognising harmful content) than on deeper emotional regulation or conduct [138,139].	Digital literacy can enhance awareness and specific skills, but knowledge gains are often partial and short-lived. Without repeated delivery and integration into whole-school practice, these programmes are unlikely to produce durable changes in how young people navigate digital environments [92].
**Promote resilience and reduce cyberbullying and related harms**	Evaluations of anti-cyberbullying curricula such as Safe Surfing, alongside broader reviews, indicate small, short-term, and non-universal improvements in bullying-related outcomes [138,139]. A meta-analysis of anti-cyberbullying programmes across school levels suggests only modest overall effects, with substantial variability between interventions and contexts [140]. Overall, whole-school bullying programmes reduce bullying by only around 15%, leaving many students still victimised despite intervention [141].	Digital literacy and anti-bullying curricula should not be treated as a stand-alone solution to cyberbullying. Their modest average effects underline the need for broader structural measures (school climate, supervision, platform design, and regulation), rather than relying on classroom programmes to “fix” complex social and technological drivers of harm.
**Encourage healthy digital habits and self-regulation; provide an ‘evidence-based’ response to online risk**	Many digital literacy and bullying-prevention programmes are short-term, rely heavily on self-report, and have limited follow-up, making the depth and durability of learning uncertain [137,138]. Stronger effects are observed when teachers and parents are actively involved, yet such models are resource-intensive and not consistently implemented. Education systems also have a history of embracing appealing interventions with weak or modest evidence: for example, large-scale reviews show growth mindset interventions have “null or very modest” effects on achievement despite widespread adoption [142], and expert commentary has raised concerns that some bullying interventions (e.g., generic bystander training) may be ineffective or even risky [141].	Digital literacy programmes risk offering a “false promise” if presented as sufficient to ensure online safety or healthy development. They should be one layer within a multi-component strategy that also includes regulation, safer platform design, parental guidance, and attention to school climate. Schools and policymakers should look beyond the label “evidence-based,” scrutinise the strength and duration of effects, and collect local outcome data before assuming meaningful impact.
**Discourage unrealistic beauty standards and expectations of body image; improve wellbeing through social media literacy**	The SoMe social media literacy programme (four sessions) aimed to improve body image and wellbeing by challenging appearance ideals and promoting critical engagement with online content. Although it produced some improvements in body image-related outcomes, it did not yield meaningful changes in self-esteem or depression at follow-up, and a subset of students reported increased pressure to attain a more muscular appearance [143].	Even well-designed literacy programmes targeting body image and wellbeing may have limited or mixed impacts, and can generate unintended consequences for some groups. This underscores the need for careful monitoring of differential effects, attention to individual vulnerabilities, and caution in presenting such programmes as robust mental-health interventions.

## Data Availability

Not applicable.
